# The Norwich Collection of Calculi

**Published:** 1888-03-03

**Authors:** 


### The Norwich Collection of Calculi.

*(Concluded from page 325.)*

The inquiry which will naturally suggest itself to the reader of this brief notice of a most interesting collection is, "Why should Norfolk be so peculiarly the home of this miserable disease of stone?" The question is far more easily asked than answered. These are facts beyond dispute, but a search for the causes leads to very debatable ground, and opens out problems which the wisest and most experienced surgeons have not been able satisfactorily to solve. No English county stands so high as Norfolk in the table of mortality from stone. The deaths, indeed, are about one in every forty-two thousand of the population. Even in London, whence come sufferers from all parts not only of Great Britain but of the world, for treatment, the deaths are only about one in seventy thousand. It was at one time believed—and held by no less an authority than the late Mr. Crosse, that the prevailing north and east winds had something to do with the prevalence of stone by checking cutaneous secretion and deranging delicate digestion, so casting additional work on the kidneys and liver; further observation has proved, however, that fewer cases come from the coast, where the winds are the keenest, than from the interior. Besides, in several very exposed countries, such as the North of Scotland, Sweden, and Norway, stone is rare; whilst in hot climates, like India, and in some parts of China, it is very common. Prout used to contend that the Norfolk dumpling was a not unimportant factor in the production of stone, by impairing the digestion; but there does not seem to be much ground for impeaching that not-to-be-despised compound. Dietary, however, has probably something to answer for. The poorer classes, in the agricultural districts specially, are great beer drinkers, but otherwise live largely on bread, cheese, very little meat except bacon and pork, and, above all, very little milk. That this latter circumstance is important in its bearing on the question is proved by the fact that cases of stone are rarely found in the children of the well-to-do classes, but very frequently in the children of the poor; in fact, Mr. Cadge, who speaks from a larger experience than almost any other surgeon, says that "stone in the young children of the poor is so common as to constitute more than half the whole number of cases." The same authority has affirmed "that strict inquiry shows that the abundance of stone in children of urban over rural populations, and of one district over another, will be found in strict accordance with the difficulty of procuring milk." He adds, as corroborating this view, that, "A few years ago, after removing a stone from a child of well-to-do parents, I was remarking to one of my assistants that this was the first instance in my practice, and that I attributed the general absence of stone to milk; the mother volunteered the statement that, in a large family, this was her only child who never could take milk, and who therefore never had any." There can be little doubt that to this cause is attributable many of the diseases which attend the early years of childhood in rural districts, and of which the Norwich Hospital has had ample experience. If the scarcity of milk, where it ought to be abundant, is an artificial cause, it may be hoped that the large attention which is now being bestowed by the Legislature on the wants of the agricultural poor, will speedily remove it. Then, again, the beer-drinking capacity of the Norfolk peasant is probably one cause of stone so frequently occurring amongst the adults of that class. The hardness of the Norfolk drinking water, coupled with the fact that the county is about the centre of the great South-Eastern chalk formation, may probably be also a powerful factor in promoting lithuric deposits. Heredity may have some bearing on the facts. In favour of this view there is considerable historical testimony. Montaigne and his father both died of stone. Sir Robert Walpole and his brother Horace (both residents in Norfolk) were afflicted with the disease, as was also their mother. The question is still an open one; but its solution is assisted by such a comprehensive and carefully-noted collection of cases as is preserved in the Norwich Museum.

